# Optimal complementary feeding practices among caregivers and their children aged 6–23 months in Kisoro district, Uganda

**DOI:** 10.1186/s40795-022-00581-0

**Published:** 2022-08-16

**Authors:** Tracy Lukiya Birungi, David Livingstone Ejalu

**Affiliations:** 1 P.O. Box 3023, Kampala, Uganda; 2 Faculty of Health Sciences, Uganda Martyrs University, Rubaga, P.O. Box 5498, Kampala, Uganda

**Keywords:** Optimal Complementary Feeding Practices, Caregivers, Children

## Abstract

**Background:**

There are many risk factors for stunting, and studies most often corroborate complementary feeding practices as a significant risk factor. Information on the prevalence of optimal complementary feeding practices and factors that lend to caregivers meeting their requirements in Kisoro district, a district with high stunting rates, is mostly lacking.

**Methodology:**

An analytical cross-sectional study that used secondary data from a USAID-funded project. Entries for 384 caregivers of children aged 6–23 months in Kisoro district were abstracted from the project database. The data was analysed using SPSS version 20. The association between independent factors and optimal complementary feeding practices was determined using multivariable logistic regressions at the three levels of the Socio-Ecological Model.

**Findings:**

Although 95% of the infants were introduced to semi-solid foods promptly, their diet was nutritionally inadequate as evidenced by the low minimum dietary diversity of 4.43%. Some of the key covariates associated with these outcomes included, the type of occupation (AOR = 21.21; CI = 2.03—221.26; *p* = 0.011), community groups (AOR = 0. 43; CI = 0.22—0.83; *p* = 0.012), not being married (AOR = 13.25; CI = 1.76—100.25; *p* = 0.012), age of the child (AOR = 2.21; CI = 1.1—4.45; *p* = 0.026); among others.

**Conclusion:**

The prevalence of MAD and MDD was very low in Kisoro district, even compared to national figures, putting these children at a very high risk of stunting. Increased advocacy is needed to support the community-level implementation of the IYCF guidelines.

**Supplementary Information:**

The online version contains supplementary material available at 10.1186/s40795-022-00581-0.

## Introduction

Stunting, a sub-form of malnutrition responsible for 14% of global childhood deaths, also heavily debilitates cognitive growth and physical capabilities, leaving a person's future less gainful than it would otherwise have been [1, 2]. There are many risk factors for stunting, and studies most often corroborate complementary feeding practices as a significant risk factor [2, 3]. Unfortunately, in many countries across the world, few children receive nutritionally adequate and safe complementary foods. Less than a quarter of the world’s population of infants aged 6–23 months meet the Minimum Acceptable Diet (MAD) criteria appropriate for their age [4]. These reports of inappropriate and inadequate complementary feeding practices may be responsible for the world's 21.9% of stunted children [5].

Several studies have reported on the inadequacy of Infant and Young Child Feeding (IYCF) practices and, consequently, complementary feeding practices. These studies advocate for the need to support populations, especially in low- and middle-income countries such as Uganda, to adopt the recommended evidence-based guidelines when practising complementary feeding [1, 5–9]. These studies have revealed that proportions of children meeting requirements for optimal complementary feeding were generally low with minimum acceptable diet (MAD), minimum dietary diversity (MDD) and minimum meal frequency (MMF) in order of lowest to slightly higher across all the regions. However, other indicators such as continued breastfeeding (CBF) at one year and introduction of solid, semi-solid or soft foods (ISSSFs) showed higher proportions for children aged 6–23 months [5, 10–12].

In Uganda, the percentages of children between 6 – 23 months of age that got a MAD, received meals the minimum number of times and had an adequately diverse diet were low at 15%, 42% and 30%, respectively [10]. Limited data exists at the district level in Uganda, although literature revealed the prevalence of MDD in Kisoro district at 3.9% [13]. Studies have documented various factors associated with complementary feeding practices, such as the caregiver's occupation type [14–17], household size [18–21], age of the caregiver [14, 15, 22, 23] and several others. This study concentrated on factors according to the Socio-Ecological Model (SEM) at three levels; settings, interpersonal and intrapersonal levels.

However, to our knowledge, none of the studies have determined the prevalence of more than three complementary feeding practices and the factors that lend to caregivers meeting the requirements for the optimal complementary feeding practices in Kisoro district. Cognizant of this empirical gap, this study sought to determine the prevalence and factors associated with the optimal complementary feeding practices (OCFPs) of caregivers and their children aged 6–23 months in Kisoro district. The findings from this study will provide evidence for use by policymakers and organisations towards strengthening existing strategies tackling the malnutrition scourge, at least in the Kigezi region.

## Methods

### Study design, area, and population

The study adopted a cross-sectional analytical design and employed quantitative data collection and analysis methods. The secondary data used was initially collected in Kisoro district, by the USAID-funded project Uganda's Integrated Community, Agriculture, and Nutrition (ICAN) Activity, from households with children aged 6–23 months; the primary respondents were the caregivers of the children. The researchers purposively selected Kisoro district because it is one of the districts in the Kigezi region, a region that has persisted with levels of stunting higher than the national levels [24]. Kisoro district is located in the South-Western part of Uganda, bordered in the North by Kanungu district, Kabale district to the East, the Republic of Rwanda to the South and the Democratic Republic of Congo to the West. The district has a total land area of roughly 729.2 km^2^ (662 km^2^ of open land, and the rest is open water and swamps). The district is approximately 510 km from Kampala, the capital city of Uganda [24, 25].

### Sample size and sampling procedure

The study used Cochran's popular formula for sample size determination [26]. The inputs were 95% confidence level and 5% margin of error, and because of the unknown proportion of children aged 6–23 months, we used maximum variability of 50%, i.e., 0.5. The sample size calculated was 384.

Multi-stage sampling was applied to select the entries for analysis from the database obtained from the USAID/ICAN project. First, the researcher chose 12 parishes from the 22 where USAID/ICAN carried out its activities using random sampling and then randomly selected 24 villages from the 12 parishes. Finally, systematic sampling was applied, which involved picking 16 households from each village by choosing the 4^th^ household till 384 were obtained. Ultimately, 16 households from the 24 villages made up the 384 per the calculated sample size.

### Data analysis

The study’s primary outcome was the prevalence of optimal complementary feeding practices among caregivers and their children aged 6–23 months in Kisoro district, Uganda. The data was analysed using SPSS version 20. The researcher determined the prevalence of five complementary feeding indicators, ISSSFs, CBF, MMF, MDD, and MAD and the factors associated with each indicator at the settings, interpersonal and intrapersonal levels of the SEM. Univariate analysis was performed to provide descriptive statistics of the indicators and factors at each level of the SEM. For bivariate and multivariate analysis, logistic regression models were run to determine the factors significantly associated with optimal complementary feeding practices (OCFPs). The procedure involved employing a stepwise backward approach to construct the models. First, all the independent variables were run separately against the dependent variable to determine Crude Odds Ratios (CORs) at a 95% confidence interval (CI). Secondly, the independent variables with a p-value < 0.05 were retained for the final model and run together against each dependent variable to obtain Adjusted Odds Ratios (AORs) at 95% CI. Variables with a p-value < 0.05 were considered statistically significant for association with OCFPs.

## Results

### Characteristics of the study population

Of the sample of 384 caregivers, 279 (72.7%), worked in the agricultural sector. More than half of the caregivers, 242 (63%) subscribed to the project livelihoods groups. Most of the caregivers lived in small households (HHs) 275 (71.6%). Few of the caregivers, 45 (11.7%), had more than two children under the age of 5 years in their care (1.61 ± 0.73). Most caregivers, 305 (79.4%), were married. More than half of the caregivers possessed more than 29 years of age, 231 (60.2%) on average 36.92 ± 15.54 years. Most of the caregivers were female, 327 (85.2%). About half of the caregivers, 201 (52.3%), had attended primary school. Over seventy per cent (73.2%) had not attended antenatal care (ANC). More than half of the caregivers (66.4%) resided in HHs where the HH head was male. Table 1

**Table 1:** Characteristics of the study population at settings, interpersonal and intrapersonal levels

Variables (*N* = 384)	Categories	Frequency (N)	Percentage (%)
**Settings Level**			
Type of occupation	working in the agricultural sector	279	72.75%
	owns non-agricultural business	35	9.1%
	formal employment	33	8.6%
	others	22	5.7%
	none	15	3.9%
Time to reach a health facility	5—30 min	150	39.1%
	> 30 min	234	60.9%
Community Groups belonged to	Livelihood group	242	63%
	^a^MIYCAN group	142	37%
**Interpersonal Level**			
Number of HH members	Small ≤ 6	275	71.6%
	Large ≥ 7	109	28.4%
Number of children under five years	≤ 2 children	339	88.3%
	> 2 children	45	11.7%
Marital status	Married	305	79.4%
	^a^Others	79	20.6%
Functional Latrine available	Yes	374	97.4%
	No	10	2.6%
Rating of WASH Situation	Best	170	44.3%
	Good	65	16.9%
	Bad	38	9.9%
	Terrible	111	28.9%
**Intrapersonal Level**			
Age of caregiver	≤ 29 years of age	153	39.8%
	> 29 years of age	231	60.2%
Sex of the caregiver	Female	327	85.2%
	Male	57	14.8%
Education status	No education	78	20.3%
	Primary school	201	52.3%
	Secondary school	80	20.8%
	University and others	25	6.5%
Attended ANC	Yes	103	26.8%
	No	281	73.2%
Sex of HH head	Female	129	33.6%
	Male	255	66.4%
Age of child	6–11 months	130	33.9%
	12–17 months	107	27.9%
	18–23 months	147	38.3%
Sex of child	Female	327	85.2%
	Male	57	14.8%
Received Nutrition education	Yes	147	38.3%
	No	237	61.7%

^a^ Others (single, divorced, separated, or widowed), MIYCAN (Maternal, Infant, Young Child, Adolescent and Newborn)

### Food groups consumed by children across the different age categories

Amongst the food groups, grains, roots and tubers were consumed by the majority of the children, 359/384 (93.5%), while eggs were the least consumed at 23/384 (6%), as shown in Table 2. Across the 6–11, 12–17- and 18–23-month age categories, grains, roots, and tubers were the most consumed (86.2%, 95.3% and 98.6%). However, for the least consumed food group, the 6–11 and 12–17 age groups had consumed eggs the least at 6.2% and 5.6%, respectively, while the 18–23 age group had consumed Vitamin-A rich fruits and vegetables the least at 2.0% as shown in Table 2.

**Table 2:** Percentage distribution of food groups consumed by children across the different age categories (*N* = 384)

Food Groups	Age Categories			Total
	6-11 months	12-17 months	18-23 months	
Grains, roots & tubers	112 (86.2%)	102 (95.3%)	145 (98.6%)	359 (93.5%)
Legumes & Nuts	21 (16.2%)	31 (28.97%)	30 (20.4%)	82 (21.4%)
Milk & milk products	27 (20.8%)	13 (12.2%)	13 (8.8%)	53 (13.8%)
Flesh foods	31 (23.9%)	37 (34.6%	35 (23.8%)	103 (26.8%)
Eggs	8 (6.2%)	6 (5.6%)	9 (6.1%)	23 (6%)
Vitamin-A rich fruits and vegetables	13 (10.0%)	11 (10.3%)	3 (2.0%)	27 (7%)
Other fruits and vegetables	50 (38.5%)	55 (51.4%)	81 (55.1%)	186 (48.4%)

### Prevalence of the optimal complementary feeding practices

Table 3 shows the prevalence of each complementary feeding indicator for the caregivers of infants 6–23 months of age in Kisoro district. Of the five indicators, 95.06% (77/81) of the caregivers timely introduced solid, semi-solid, or soft foods and 84.81% (67/79) of mothers of infants 12 to 15 months of age had continued breastfeeding their children till one year. Results also showed that 76.56% (294/384) of infants 6 to 23 months met the minimum meal frequency standards (MMF). However, the Minimum Dietary Diversity (MDD) and Minimum Acceptable Diet (MAD) were too low each at 4.43% (17/384) for infants 6 to 23 months of age.

**Table 3:** Prevalence of each complementary feeding indicator for the caregivers of infants 6–23 months of age in Kisoro district

CFI	ISSSFs	CBF	MDD	MMF	MAD
Prevalence	95.06%	84.81%	4.43%	76.56%	4.43%

### Factors associated with optimal complementary feeding practices

As seen in Table 4, the type of occupation and community group were associated with MMF at the settings level. Caregivers that gained income in other ventures such as begging were 21 times more likely to meet the MMF requirements for their children than those working in the agricultural sector (AOR = 21.21; CI = 2.03—221.26; *p* = 0.011). Caregivers in the livelihood group were 57.4% less likely to achieve minimum meal frequency for their children than those in the MIYCAN group (AOR = 0. 43; CI = 0.22—0.83; *p* = 0.012).

**Table 4:** Factors Associated with Optimal Complementary Feeding Practices of Caregivers and their Children Aged 6–23 Months in Kisoro District, Uganda (*N* = 384)

SEM Domain	Independent Variable	Categories	COR (95%CI)	AORs (95%CI)	*p*-value	Indicator
**Settings**	Type of occupation	working in the agricultural sector	1	1	0	MMF
		owns non-agricultural business	1.31 (0.55—3.14)	3.01 (0.95—9.55)	0.061	
		formal employment	10.51 (1.41—78.38)	2.08 (0.49—8.86)	0.324	
		others	0.29 (0.10—0.82)	1.21 (0.25—5.84)	0.814	
		none	1.12 (0.4—3.14)	21.21(2.03- 221.26)	0.011*	
	Community group	MIYCAN group	1	1	0	
		Livelihood group	0.44 (0.25—0.75)	0.43 (0.22—0.83)	0.012*	
**Interpersonal level**	Marital status	Married	1	1	0	CBF
		Others	0.05(0.01—0.34)	13.25 (1.76—100.25)	0.012*	
	Number of HH members	≤ 6 members	1	1	0	MDD, MAD
		≥ 7 members	3.00(1.13—8.00)	0.35 (0.13—0.94)	0.037*	
**Intrapersonal level**	Age of caregiver	≤ 29 years	1	1	0	ISSSFs
		> 29 years	0.54 (0.33—0.89)	1.73 (1.04—2.88)	0.035*	
	Age of child	6–11 months	1	1	0	MMF
		12–17 months	0.42(0.21—0.82)	2.21(1.1—4.45)	0.026*	
		18–23 months	0.3 (0.16—0.56)	0.94 (0.49—1.78)	0.846	

^*^ significant when *p* < 0.05

At the interpersonal level, the study found the number of HH members associated with MDD and MAD, while marital status was associated with CBF. Caregivers in large HHs (seven or more HH members) were 65.4% less likely to offer their children an acceptable and diverse diet than their counterparts in smaller HHs (6 or fewer HH members) (AOR = 0.35; CI = 0.13—0.94; *p* = 0.037). Caregivers who were not married were 13.25 times more likely to have continued breastfeeding their children by one year of age than their unmarried counterparts (AOR = 13.25; CI = 1.76—100.25; *p* = 0.012).

At the intrapersonal level, the caregiver's age was associated with ISSSFs, while the child's age was associated with MMF. Caregivers older than 29 years were 1.7 times more likely to introduce solid, semi-solid, and soft foods timely to their children than their younger counterparts (AOR = 1.73; CI = 1.04—2.88; *p* = 0.035). Caregivers for children aged between 12–17 months were 2.2 times more likely to meet the MMF requirements for their children than caregivers with children in the 6–11 months age bracket (AOR = 2.21; CI = 1.1—4.45; *p* = 0.026).

## Discussion

Of the 81 caregivers with children 6–8 months of age, 95.06% introduced solid, semi-solid or soft foods in a timely fashion, relatively higher than the national prevalence, which stood at 78.8% [10]. However, a study in China amongst persons in rural settings, comparable to this study, obtained similar findings where 95. 4% of the infants aged 6–8 months received solid, semi-solid or soft foods appropriately [27].

Minimum Dietary Diversity was too low at 4.43%, a proportion significantly lower than that for Uganda at 30% [10]. The MDD was exceedingly low, similar to other studies done after the COVID-19 pandemic traversed the globe. Kundu et al.[28] and Minja et al.[29] explained that MDD was heavily associated with the earning potential of HHs and in rural areas where most HHs earned low incomes, and in the presence of the pandemic that negatively impacted income earning, MDD was, in turn, very low. Amongst the food groups, grains, roots and tubers were consumed by most children (93.5%), while eggs were the least consumed at 6%. Similarly, a study carried out in Northern Uganda also found that cereals were more consumed than foods of animal origin, which the people attributed to grains being cheaper to purchase and easier to access than foods of animal origin [3].

The study findings revealed that at the settings level, caregivers that gained income in non-formal or agricultural ventures such as begging and remittances, among others, were more likely to meet the requirements for MMF for their children compared to caregivers working in the agricultural sector. Similarly, studies carried out in Poland, Austria, and Southern Benin reported that parents or caregivers, particularly mothers who were employed or heavily involved in income-generating activities, were less likely to have their children meet the MMF requirements [18, 29]. Our study findings further indicate that caregivers who belonged to village livelihood groups were less likely to achieve minimum meal frequency for their children than those in the MIYCAN group. This is because caregivers in the livelihood group were more involved in income-generating activities, which decreased the time to care for the children and hampered meeting requirements for MMF [18, 29]. On the other hand, caregivers in the MIYCAN groups regularly received nutrition training, a factor documented to favour meeting the requirements of the optimal complementary feeding practices [15, 23].

At the interpersonal level, caregivers in large HHs were less likely to offer their children an acceptable and diverse diet than those living in small HHs. Studies in Ethiopia, Australia, and Benin also concluded that HHs with more than seven residents usually had sub-optimal complementary feeding practices because of the food insecurity brought on by income limitations; the low incomes cannot ably support the large household sizes [18–20]. In addition, unmarried caregivers were more likely to have continued breastfeeding their children by one year than married caregivers. This finding differed from what studies in Kenya, and Southern Benin discovered, where married caregivers were more likely to meet the requirements for OCFPs [18, 30]. However, Scott et al. [31] concluded that partners could be deterrents to continued breastfeeding, and mothers that did not have partners would breastfeed longer because they had the time to dedicate to their children and also issues such as sagging breasts and losing the interest of your partner were not a worry [32] promoting longer durations of breastfeeding.

At the intrapersonal level, caregivers older than 29 years were more likely to promptly introduce solid, semi-solid, and soft foods to their children than their younger counterparts. Other studies with similar findings concluded that a mother's age was a predictor for complementary feeding practices because older mothers were more experienced and knowledgeable in taking care of their children compared to their younger, less knowledgeable counterparts [15, 23]. Caregivers for children aged between 12–17 months were more likely to meet the MMF requirements for their children compared to caregivers with children in the 6–11 months age bracket. A study with similar findings explained that children within the former age bracket had likely settled into the pattern of the family meals, which were the prescribed number of times a child that age should be fed, lending to meeting the MMF requirements [33].

### Recommendations

Increased advocacy by nutrition champions and organisations for augmented funding to the nutrition sector; to support the direct implementation of the IYCF guidelines and Multisectoral Nutrition Action Plans. The government and policymakers must strengthen existing initiatives and develop new intervention measures to increase the citizenry's socioeconomic position, education status, and occupational opportunities for better CFPs. More research in other regions of the country on factors associated with IYCF.

## Conclusion

The prevalence of Minimum Acceptable Diet (MAD) and Minimum Dietary Diversity (MDD) was very low in Kisoro district, putting these children at a very high risk of becoming stunted. At all levels of the SEM, there was at least a factor associated with OCFPs intimating the need for all sectors to work together to break down barriers preventing caregivers from meeting requirements for OCFPs as we forge forward to end malnutrition for all.

### Study limitations

The study utilised secondary data collected in Kisoro district, so the findings may not be ably generalised to other districts not in the Kigezi region. In addition, the dataset provided to the study was from rural communities, and the findings may not translate to urban communities.

For Operational Definitions see Additional File 3

## Supplementary Information

**Additional file 1. **

**Additional file 2. **Results of the univariate analysis of the complementary feeding practices.

**Additional file 3. **Operational definitions.

## Data Availability

The dataset supporting the conclusions of this article is included as Additional File 1.

## Abbreviations

ANC: Antenatal Care
AORs: Adjusted Odd Ratios
CBF: Continued breastfeeding
CORs: Crude Odd Ratios
HH: Household
ISSSFs: Introduction to Solid, Semi-solid, and Soft Foods
IYCF: Infant and Young Child Feeding
MAD: Minimum Acceptable Diet
MDD: Minimum Dietary Diversity
MIYCAN: Maternal, Infant, Young Child, Adolescent, and New-born
MMF: Minimum Meal Frequency
OCFPs: Optimal Complementary Feeding Practices
WHO: World Health Organisation
