# Supplementary material for: Optimal complementary feeding practices among caregivers and their children aged 6–23 months in Kisoro district, Uganda
Source: BMC Nutr. 2022 Aug 16;8:81. doi: 10.1186/s40795-022-00581-0 (PMC9379229; doi:10.1186/s40795-022-00581-0)
Supplement: Supplementary file 2 — Additional file 2. Results of the univariate analysis of the complementary feeding practices. [file 40795_2022_581_MOESM2_ESM.docx]

**Results of the Univariate Analysis of the Complementary Feeding Practices**

| **Complementary Feeding Practice** | **Frequency** | **Percent** |
| --- | --- | --- |
| ISSSFs (n= 81) | 77 | 95.06% |
| CBF (n=79) | 67 | 84.81% |
| MDD (n=384) | 17 | 4.43% |
| MMF (n=384) | 294 | 76.56% |
| MAD (n=384) | 17 | 4.43% |
