# Supplementary material for: Optimal complementary feeding practices among caregivers and their children aged 6–23 months in Kisoro district, Uganda
Source: BMC Nutr. 2022 Aug 16;8:81. doi: 10.1186/s40795-022-00581-0 (PMC9379229; doi:10.1186/s40795-022-00581-0)
Supplement: Supplementary file 3 — Additional file 3. Operational definitions. [file 40795_2022_581_MOESM3_ESM.docx]

**Operational Definitions**

***Optimal complementary feeding practices (OCFPs):*** This is the feeding of other foods and liquids to a breastfeeding child aged 6 to 23 months that meets the requirements for introduction to solid, semi-solid and soft foods, continued breastfeeding at one year, minimum dietary diversity, minimum meal frequency and minimum acceptable diet.

***Minimum dietary diversity (MDD):*** This is defined and measured as the proportion of children aged 6-23 months who received foods from 4 or more food groups consisting of; grains, roots and tubers; legumes and nuts; dairy products; flesh foods; eggs; vitamin-A rich fruits and vegetables; and other fruits and vegetables the previous day or 24 hours.

***Minimum meal frequency (MMF):*** the proportion of breastfed children who receive solid, semi-solid or soft foods the minimum number of times or more; two times for infants 6-8 months and three times for children 9-23 months.

***Introduction of solid, semi-solid or soft foods (ISSSFs):*** This indicator measured the timeliness of the introduction of complementary foods and is defined as the proportion of children who received solid, semi-solid or soft foods at 6-8 months of age.

***Minimum Acceptable Diet (MAD)****:* the proportion of children aged 6 - 23 months who consumed the minimum dietary diversity and minimum meal frequency in the previous 24 hours.

***Setting****:* This is a location (workplaces, place of residence, closeness to a health facility) or social context (community groups) in which people carry out everyday activities that influence how they practice complementary feeding.

***Community group:*** This is defined as five or more people who live in the same area and either work together to maximise economic opportunities from agriculture and non-agriculture-based livelihoods through connections to markets and financial services (livelihood group) or participate in activities such as nutrition education to reduce malnutrition in mothers, infants, young children, adolescents, and new-borns (MIYCAN group).

***WASH (Water, Sanitation and Hygiene) Situation:*** Possession of a functional latrine and handwashing facility (with a cleansing agent and water) gauged as best, good, bad and terrible. For best, the households had a functional latrine and handwashing facility with water and a cleansing agent; for good, the households had a functional latrine and handwashing facility with/without water and a cleansing agent or not. For bad; may or may not possess a functional latrine and handwashing facility with/without water and a cleansing agent or not. For terrible; may or may not possess either a functional latrine or handwashing facility lacking water or a cleansing agent.

***Caregiver:*** A parent (mother or father) or any other person directly involved in meeting the child's needs (feeding, grooming, among others) and lives within the household.
